# Early detection of pemetrexed-induced inhibition of thymidylate synthase in non-small cell lung cancer with FLT-PET imaging

**DOI:** 10.18632/oncotarget.12085

**Published:** 2016-09-16

**Authors:** Xiao Chen, Yizeng Yang, Ian Berger, Urooj Khalid, Akash Patel, Jenny Cai, Michael D. Farwell, Corey Langer, Charu Aggarwal, Steven M. Albelda, Sharyn I. Katz

**Affiliations:** ^1^ Department of Radiology, University of Pennsylvania Perelman School of Medicine, Philadelphia, PA, USA; ^2^ Department of Radiology, Institute of Surgery Research, Daping Hospital, Third Military Medical University, Chongqing, China; ^3^ Department of Medicine, University of Pennsylvania Perelman School of Medicine, Philadelphia, PA, USA

**Keywords:** FLT, PET, pemetrexed, lung cancer, flare

## Abstract

Inhibition of thymidylate synthase (TS) results in a transient flare in DNA thymidine salvage pathway activity measurable with FLT ([^18^F]thymidine)-positron emission tomography (PET). Here we characterize this imaging strategy for potential clinical translation in non-small cell lung cancer (NSCLC). Since pemetrexed acts by inhibiting TS, we defined the kinetics of increases in thymidine salvage pathway mediated by TS inhibition following treatment with pemetrexed *in vitro*. Next, using a mouse model of NSCLC, we validated the kinetics of the pemetrexed-mediated flare in thymidine salvage pathway activity *in vivo* using FLT-PET imaging. Finally, we translated our findings into a proof-of-principle clinical trial of FLT-PET in a human NSCLC patient. In NSCLC cells *in vitro*, we identified a burst in pemetrexed-mediated thymidine salvage pathway activity, assessed by ^3^H-thymidine assays, thymidine kinase 1 (TK1) expression, and equilibrative nucleoside transporter 1 (ENT1) mobilization to the cell membrane, that peaked at 2hrs. This 2hr time-point was also optimal for FLT-PET imaging of pemetrexed-mediated TS inhibition in murine xenograft tumors and was demonstrated to be feasible in a NSCLC patient. FLT-PET imaging of pemetrexed-induced TS inhibition is optimal at 2hrs from therapy start; this timing is feasible in human clinical trials.

## INTRODUCTION

Successful inhibition of thymidylate synthase (TS), a critical enzyme in the *de novo* thymidine synthesis pathway, is the key target of chemotherapeutics such as 5-fluorouracil (5-FU), pemetrexed, and capecitabine. Once TS is blocked, a rapid compensatory increase in the thymidine salvage pathway occurs resulting in a rapid uptake of extracellular thymidine. This burst or “flare” in uptake can be visualized using ^18^F-thymidine (FLT), an analogue of thymidine and a PET (positron emission tomography) radiotracer. FLT was first described as an imaging biomarker of thymidine salvage activity by Shields and Grierson *et al* in 1998 [1] and is a validated surrogate marker of proliferation in lung cancer [1–4]. In the cell, FLT becomes mono-phosphorylated and trapped by the key thymidine salvage pathway enzyme thymidine kinase 1 (TK1); thus tumors become more FLT-avid as thymidine salvage pathway activity increases. As such, this drug-induced salvage pathway “flare” effect provides an imaging opportunity to determine successful TS inhibition in the tumor within hours of starting therapy.

The TS-inhibition induced FLT “flare” effect appears to be mediated primarily though one or both of two mechanisms. The first is an increase in TK1 function, the rate-limiting step of the thymidine salvage pathway. This may occur either through an increase in TK1 activity [5, 6], which is modulated by its physical state [7], or protein expression of TK1[8] [9]; both of these effects are carefully modulated throughout the cell cycle [5, 10, 11]. This boost in TK1 function serves to compensate for the inhibition of the *de novo* synthesis pathway allowing continued supply of thymidine for cellular division. Increased cell surface density of equilibrative nucleoside transporter 1 (ENT1) may also contribute to the FLT “flare”. This may occur either from ENT1 mobilization to the cell surface [12] or an increases in ENT1 expression [6]. ENT1 transport is regulated by the cell cycle and is the dominant mechanism of increased FLT entry for proliferating cells [13–15]. In some studies ENT1 has been shown to rapidly mobilized to the cell surface within hours of successful TS-inhibition [12, 16] while others have failed to observe this shift in ENT1 distribution [5].

It is still uncertain whether this FLT “flare” imaging technique can be a reliable predictor of tumor response to therapy. A recent clinical pilot study of FLT “flare” as a measure of response to therapy with pemetrexed-based therapy in NSCLC showed no association between the presence of the FLT “flare” and clinical outcome [17]. Though this study had a small heterogenous population of patients, it does raise the need for further pre-clinical modeling to fully characterize this imaging strategy prior to clinical translation. In order to study the predictive value of this technique, it is first critical to determine the optimal timing of measurement of the “flare”. The TS-inhibitor mediated thymidine salvage pathway “flare” is a transient metabolic phenomenon which dissipates within hours and there has been variability in the reported timing of measurement of this effect from 1-48 hrs following exposure to therapy [8, 16–21]. This variability is likely due to differing mechanisms of the “flare” depending on cancer type and specific TS inhibitor therapy. We focus here on pemetrexed, a TS-inhibitor commonly used in 1^st^ line therapy for non-small cell lung cancer.

In this study, we define the kinetics of the pemetrexed-induced FLT “flare” in order to determine the optimal timing of FLT imaging for further preclinical study and ultimately translation to the clinic. Furthermore, we elucidate the mechanism of FLT “flare” following pemetrexed-induced inhibition and characterize the potential impact of concurrent therapy with a platin drug on “flare” kinetics. This is important since pemetrexed regimens typically include a DNA-damaging platin agent such as carboplatin or cisplatin. Finally, we conduct a pilot of FLT-PET imaging of pemetrexed-induced TS inhibition in a patient with NSCLC to validate the feasibility of this imaging technique at the determined optimal time point.

## RESULTS

### Pemetrexed-induced TS inhibition results in a “flare” in thymidine salvage pathway activity peaking at 2 hours *in vitro* which is partially blocked by ENT1 inhibition

**Figure 1:**
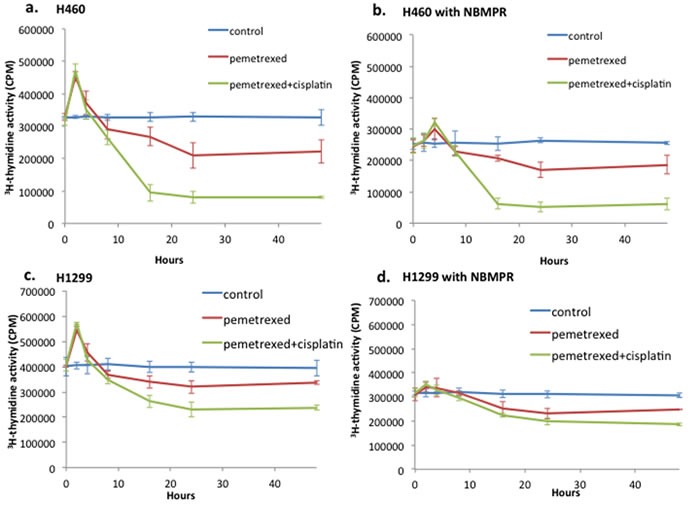
Pemetrexed-induced TS inhibition results in a “flare” of the thymidine salvage pathway activity ^3^H-thymidine assay was performed on PEM-sensitive NSCLC H460 in untreated control (culture medium only), pemetrexed (100nM) and combination therapy with pemetrexed (100nM) plus cisplatin (10mM). A “flare” of thymidine salvage pathway activity peaked at 2 hours of pemetrexed therapy exposure in both the H460 **a**. and H1299 **c**. NSCLC cell lines. This “flare” in thymidine salvage pathway activity was blunted by pretreatment of cell cultures with ENT1 inhibitor NBMPR in both H460 **b**. and H1299 **d**. NSCLC cell lines.

Initially, we sought to define the kinetics the TS inhibition-induced “flare” of the thymidine salvage pathway in NSCLC cells *in vitro*. H460 and H1299 NSCLC lines, both pemetrexed sensitive (Supplemental data Figure S2), demonstrated a significant transient compensatory “burst” in thymidine salvage pathway activity peaking at 2 hours as measured on ^3^H-thymidine assays (Figure 1) relative to untreated controls. The average magnitude of the thymidine salvage pathway “flare” at 2 hours was 38% above baseline in H460 and 35% over baseline in H1299. The addition of cisplatin to pemetrexed treatment of these cells did not impact the amplitude or timing of the thymidine salvage pathway “flare” effect (H460: *p* = 0.32; H1299: *p* = 0.12).

**Figure 2:**
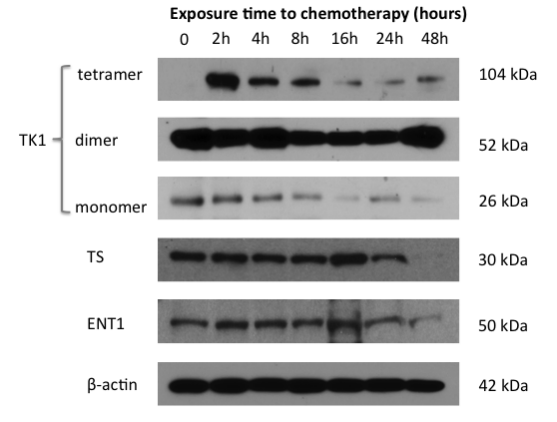
Induction of tetramer state of TK1 corresponds temporally with pemetrexed-induced thymidine salvage pathway “flare.” A time course of exposure of PEM-sensitive NSCLC cell line H460 to combination therapy with PEM/cisplatin *in vitro* revealed induction of highly activated tetramer TK1 state corresponding temporally to the FLT “flare” observed at 2 hours. ENT1 and TS protein levels remain unchanged in the “flare” period. All protein levels slowly decreased beyond 8 hours due to successful cell cycle inhibition by PEM/cisplatin therapy.

In order to determine if intact ENT1 function was required for this pemetrexed-induced DNA salvage pathway “flare”, pemetrexed-induced changes in DNA salvage pathway activity was also examined in the presence of ENT1 inhibition. When pre-treated with ENT1 inhibitor NBMPR (Figure 1), the pemetrexed-induced “flare” in DNA salvage pathway was markedly diminished in both the H460 and H1299 NSCLC cell lines. In the H460 and H1299 cell lines, the magnitude of the pemetrexed-induced “flare” at 2hrs was 3.2% and 7.3% over the baseline respectively. With the addition of cisplatin to pemetrexed, there was no significant change in the blunting of the DNA salvage pathway flare observed in the presence of ENT1 inhibition. For H460 and H1299 cells treated with the combination of pemetrexed and cisplatin, the magnitude of the pemetrexed-induced DNA salvage pathway flare at 2 hours was 2.8% and 11.87% over baseline respectively. The addition of cisplatin to pemetrexed treatment of these cells did not impact the amplitude or timing of the thymidine salvage pathway effect: H460: *p* = 0.96; H1299: *p* = 0.34).

By 24 hours of therapy, both cells treated with pemetrexed and cells treated with a combination therapy of pemetrexed and cisplatin exhibited a significant suppression of proliferation relative to untreated controls. For cells treated with pemetrexed alone, intracellular ^3^H-thymidine accumulation was decreased by 36% and 19% at 24 hours of exposure relative to baseline for H460 and H1299 respectively. At 24 hours of exposure to combination therapy with pemetrexed and cisplatin, intracellular ^3^H-thymidine accumulation decreased by 75% and 42% relative to baseline for H460 and H1299 respectively. Significantly greater suppression of proliferation was exhibited by the combination therapy with cisplatin and pemetrexed versus pemetrexed alone (H460: *p* = 0.007; H1299: *p* = 0.016). Thus, for pemetrexed alone and in combination with cisplatin, the “flare” effect was optimal *in vitro* at 2 hours after starting therapy. These findings were validated in 6 additional human NSCLC cell lines demonstrated to be sensitive to pemetrexed and cisplatin (Supplemental data Figure S2 and S3).

### Pemetrexed-induced thymidine salvage pathway “flare” is a result of a combination of redistribution of ENT1 receptors and activation of TK1 to the tetramer state

In order to gain insights into the mechanism for the pemetrexed-induced thymidine salvage pathway “flare”, the protein expression of several key components of the thymidine salvage pathway were examined following exposure to pemetrexed or pemetrexed plus cisplatin. TK1, rate-limiting enzyme of the thymidine salvage pathway, exists in a modestly active monomer, moderately active dimer and highly active tetramer state. Treatment of both H460 and H1299 cells revealed a shift in TK1 protein to the tetramer state following 2 hours of exposure to pemetrexed therapy, with an increase of TK1 in the tetramer state of 10 fold (*p* < 0.0001) and 1.6 fold (*p* = 0.011), respectively, and corresponding decreases in the monomer and dimer states of TK1 (Figure 2). These data are consistent with the timing of the pemetrexed-induced “flare” in thymidine salvage pathway activity observed at 2 hours using ^3^H-thymidine assays (Figure 1). Similar to data from the ^3^H-thymidine assay data, the induction of this tetramer state was unaffected by exposure to cisplatin (H460: *p* = 0.48; H1299: *p* = 0.70).

Levels of total TS and ENT1 protein expression were not significantly changed during the 1^st^ 8 hours of treatment. However, by 16-24 hours of exposure of cells to pemetrexed plus cisplatin, both cell lines exhibited a gradual decrease in protein expression of TK1, TS and ENT1 in keeping with successful cell cycle arrest. At 24 hours, there was widespread decrease in expression in the measured proteins relative to baseline. TK1 (total protein) was decreased by 36% (*p* = 0.0092) and 35% (*p* = 0.007), TS decreased by 38% (*p* = 0.04) and 8.67% (*p* = 0.64), and ENT1 decreased by 17% (*p* = 0.19) and 84% (*p* = 0.0005) for H460 and H1299 respectively.

**Figure 3:**
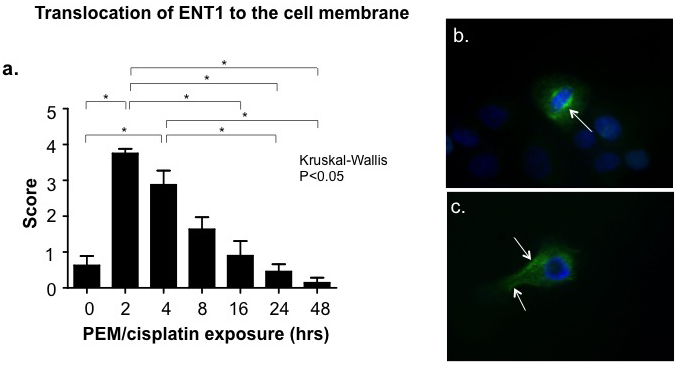
Translocation of ENT1 to the cell surface corresponds temporally with the pemetrexed-induced thymidine salvage pathway “flare.” Immunofloresence microscopy utilizing a time course of exposure of the pemetrexed (PEM)-sensitive NSCLC cell line H460 to combination therapy with PEM(100nM)/cisplatin(10 μM) *in vitro* revealed translocation of ENT1 to the cell surface from the peri-nuclear cytoplasm maximal at 2 hours of exposure to PEM or PEM/cisplatin corresponding to the timing of the FLT “flare”. **a**. Cells were scored on a scale 0 (no ENT1 translocation) to 5 (high ENT1 translocation to the cell surface) Microscopy demonstrated NSCLC staining for ENT1 (Green stain) and nuclear membrane (blue stain) following 2 hours of exposure to **b**. culture medium control **c**. or combination of pemetrexed and cisplatin. Arrows depict concentration of ENT1 staining.

In addition to examination of protein expression of key elements of the thymidine salvage pathway, we also examined subcellular localization of ENT1. By immunofloresence microscopy, we detected a transient but significant shift in subcellular localization of ENT1 protein from the peri-nuclear location to the cell surface following treatment with pemetrexed. Cell-surface localization of ENT1 peaked at 2 hours of exposure to pemetrexed with a 3.9 fold (*p* < 0.0001) and 4.2 fold (*p* < 0.0001) increase for H460 and H1299 respectively. This peak was followed by a steady shift back to peri-nuclear localization with a return to near baseline distribution by 24 hours (Figure 3).

### *In vivo* modeling validates *in vitro* kinetics of pemetrexed-induced “flare” in thymidine salvage pathway activity

**Figure 4:**
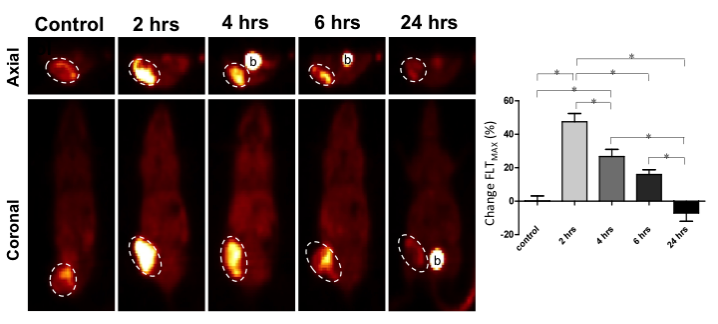
FLT-PET imaging of pemetrexed-induced TS inhibition demonstrates a FLT “flare” peaking at 2 hours in a preclinical model of NSCLC Human NSCLC tumor-bearing murine xenografts were treated with a combination of pemetrexed (PEM) and cisplatin in order to model 1^st^ line therapy. FLT-PET was performed at baseline and at multiple time points following therapy start. Tumor avidity for FLT was observed to peak at 2 hours following PEM-based therapy. By 24 hours of therapy, tumors began to demonstrate inhibition of proliferation. (b.) Excreted radiotracer within the bladder.

Prior to conducting a study of FLT flare kinetics, we confirmed efficacy of combined therapy with pemetrexed and cisplatin *in vivo*. A pilot study of 12 H460 tumor-bearing xenografts, 4 control (vehicle only) and 8 treated (combination of pemetrexed and cisplatin), revealed tumor growth inhibition of 88% in those treated with pemetrexed and cisplatin relative to vehicle only controls (Supplemental data Figure S1). Consequently, *in vivo* modeling of 1^st^ line therapy of NSCLC xenografts with combined pemetrexed and cisplatin therapy revealed a peak in tumor avidity for FLT at 2 hours of exposure to therapy (Figure 4). At 2 hours of therapy, tumor FLT_MAX_ increased 47.5% (±12.0%, *p* = 0.008) relative to baseline in the xenografts treated with cisplatin and pemetrexed but remained unchanged in vehicle treated controls (*p* = 0.37). After this post-therapeutic peak or “flare” in tumor avidity for FLT at 2 hours, tumor avidity for FLT decreased over the course of 24 hours. By 24 hours following start of combination therapy with pemetrexed plus cisplatin, tumor avidity for FLT began to fall below baseline, albeit not significantly, with a mean tumor FLT_MAX_ of -6.0% (±10.5%, *p* = 0.35). This is likely due to the impact of successful therapy on proliferation, which occurs in a similar timeframe to the *in vitro* studies of thymidine salvage pathway activity and protein expression (Figures 1 and 2).

### Human FLT-PET imaging of pemetrexed-induced “flare” at 2 hours of therapy is feasible

**Figure 5:**
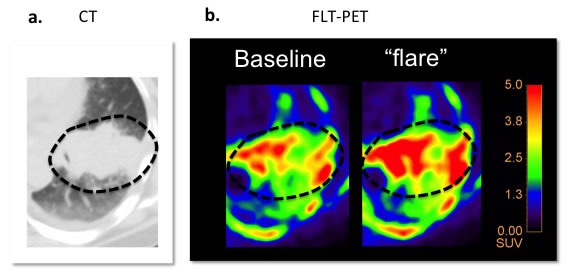
FLT “flare” in response to PEM-based therapy in a human patient These images are from a 63 year-old male with NSCLC participating in our exploratory clinical trial of FLT-PET “flare”. **a**. Baseline CT revealed a 6 cm mass in the left upper lobe. **b**. Baseline FLT-PET revealed mild avidity 3 days pre-therapy. **c**. FLT-PET “flare” imaging performed after 2 hours following administration of combination therapy with PEM and carboplatin revealed a burst in tumor avidity in keeping with the FLT “flare.”

We next sought to extend our findings to human lung cancer through a proof-of-concept clinical trial of FLT-PET/CT as a measure of TS inhibition in patients with advanced NSCLC. Baseline FLT-PET performed three days prior to start of therapy revealed a FLT-avid (SUV_MAX_ of 5.4) solid 6 cm mass in the left upper lobe in keeping with the known NSCLC. A repeat FLT-PET/CT scan performed at 2 hours following the first infusion of combined therapy with pemetrexed and carboplatin revealed a post-therapeutic increase in tumor avidity for FLT with a 44% increase in SUVmax (SUV_MAX_ of 7.8) relative to baseline (Figure 5). Thus, in a human patient with NSCLC, FLT-PET revealed a “flare” in tumor avidity for FLT at 2 hours following intravenous infusion with pemetrexed and carboplatin; this study demonstrates the feasibility of translating this technique, using a 2 hour time point, to the clinic. Nonetheless, more patients will need to be studied to validate the predictive value of this post-therapeutic FLT “flare” for lung cancer patients treated with 1^st^ line pemetrexed-based therapies.

## DISCUSSION

In this study we determine the optimal timing for imaging the pemetrexed-induced thymidine salvage pathway “flare” in NSCLC. To our knowledge this is the first detailed study of timing and mechanism of pemetrexed-induced DNA salvage pathway “flare” in NSCLC. *In vitro* and *in vivo* data in this study consistently show a transient peak in thymidine salvage pathway “flare” activity at 2 hours of exposure to pemetrexed. This timing of the thymidine salvage pathway “flare” is in the range of that reported in the literature for other TS inhibitors but earlier than the time points used in other clinical trials of TS inhibitor induced FLT “flare” [17, 18]. By optimizing the timing of the FLT “flare” in pemetrexed therapy, future studies will be positioned to determine the predictive value of this imaging strategy in this therapeutic setting. Moreover, to our knowledge, this is the first study to determine that concurrent treatment with cisplatin does not alter the timing or magnitude of the TS-inhibition mediated salvage pathway “flare.” This is critical to the utility of this technique for clinical translation of this imaging strategy since pemetrexed is frequently used in combination with a platin-based therapeutic for oncologic management.

Tumor avidity for FLT is well correlated with TK1 expression [23] and activity [10], the rate-limiting step in the thymidine salvage pathway [23], and with the cell surface expression of ENT1 [13, 14]. Analysis of protein expression in this study suggests that the pemetrexed-induced salvage “flare” effect is likely attributable, at least partially, to an increase in TK1 activity via a shift in enzyme state to highly active tetramer. Our data also indicate that ENT1 contributes to the pemetrexed-induced “flare” in DNA salvage pathway activity and that transient localization of ENT1 to the cell surface likely contributes to this thymidine salvage pathway “flare” effect. There is support in the literature for both increases in TK1 activity[5, 6, 8] and redistribution of ENT1 to the cell surface[12, 16] as potential mechanisms for the TS-inhibition mediated thymidine salvage pathway “flare” effect. The ENT1 transporters may also contribute to the subsequent decrease in tumor avidity for FLT following the “flare” as they have been demonstrated to contribute to nucleoside/nucleotide efflux from the cell[24] and to be decreased in expression as cells exit active cell cycling [13] in response to therapy.

Our data also demonstrate that the pemetrexed-induced FLT “flare” is not mediated by changes in TK1 or ENT1 protein expression. This is plausible since changes in protein expression are unlikely to occur in a 2 hour time interval. Lee *et al* described a bimodal FLT “flare” occurring at 2 hours and 24 hours in most cell lines treated with 5-FU[8]. This later 24-48 hour peak in salvage pathway “flare” may be due to increased expression of TK1 [8, 16] and/or ENT1 [6], which is observed following mono-therapy with 5-FU [6, 8] or BGC 945 [16]. We hypothesize that the nature of the therapeutics, such as the choice of TS inhibitor and use of mono-therapy versus combination therapy, may impact contributions of available salvage pathway compensatory mechanisms resulting in differing FLT “flare” kinetics. As such, we posit that our use of concurrent therapy with a DNA damaging platin agent prevents compensation of the DNA salvage pathway through increases in protein expression, seen in some models at 24-48 hours, due to overall suppression of protein expression. Of note, while ENT1 is the dominant transporter for FLT, in this study we did not evaluate the potential contributions of other cell surface transporters such as the concentrative transporters, which are known to contribute to FLT metabolism [14, 24] and may also play a role in the dynamics of pemetrexed-induced FLT “flare”.

Our study results support previously published data that the TS-inhibition induced “flare” in thymidine salvage pathway activity rapidly dissipates. Using pemetrexed therapy, the “flare” effect was dissipated by 24 hours both *in vitro* and *in vivo*. By 24 hours, the effects of the chemotherapeutics have begun to impair tumor proliferation with resulting suppression of thymidine salvage pathway activity and protein synthesis. These findings are compatible with the findings of Barthel *et al* who demonstrated that tumor avidity for FLT is significantly decreased relative to baseline by 48 hours of exposure to 5-FU [25]. This anti-proliferative effect is greater with combined therapy of pemetrexed and cisplatin compared to pemetrexed alone, in keeping with the fact that these cell lines are sensitive to both pemetrexed and cisplatin. In light of these data we propose that an imaging strategy designed to specifically detect the success of pemetrexed-induced TS inhibition should measure thymidine salvage pathway at 2 hours. In contrast, imaging designed to evaluate the impact of pemetrexed-induced effects on proliferation should measure thymidine salvage pathway activity after 24 hours of therapy. Additional preclinical modeling will be necessary, using dynamic PET imaging, to determine the potential impact of pemetrexed-based therapeutics on the relative blood bioavailability of FLT metabolites as this can impact quantitation of FLT and will be the subject of future studies.

Finally, our pilot of the FLT “flare” imaging strategy in a patient with NSCLC suggests that FLT-PET imaging at the 2 hour window following infusion with pemetrexed-based therapy is feasible in the clinic and a clear “flare” can be imaged at this time point. Further study will be necessary to determine the predictive value of this imaging strategy. It is plausible that detection of response to pemetrexed-based therapy at this early time point, months before what is currently possible by conventional assessments with computed tomography (CT), will have a benefit to the patient in terms of time saved during which tumors can grow and patients are exposed to unwarranted therapeutic toxicities. In sum, our data provide a rationale for using the 2 hour time point for studies of pemetrexed-induced FLT-PET “flare” when modeling 1^st^-line therapy in NSCLC. Future studies are needed to determine the predictive value of this imaging technique as well as the potential for benefit to the patient by clinical translation.

## MATERIALS AND METHODS

### Chemotherapeutics and imaging radiopharmaceuticals

For *in vitro* studies, pemetrexed (Santa Cruz Biotechnology, Dallas, Texas) and cisplatin (Sigma-Aldrich Corp., St. Louis, MO) were provided in solid form, dissolved in water and stored at -20°C as a 0.2 mM and 1 mM stock, respectively. For *in vivo* use, both human and murine, pemetrexed (ALIMTA; Eli Lilly and Company, Indianapolis, IN) and cisplatin (Teva Pharmaceuticals, Petach Tikva Israel) were provided freshly prepared as a 1mg/ml sterile saline solution by the Abramson Cancer Center Pharmacy. For *in vivo* murine studies, chemotherapeutics were stored at 4°C. [^18^F]FLT was produced on site in the University of Pennsylvania PET Center Cyclotron facility. [^18^F]FLT average specific activity was 5.32 +/− 2.14 Ci/umol, and radiochemical purity >99%.

### Cell lines and culture

All human non-small cell lung cancer cell lines were obtained from American Type Culture Collection (ATCC, Manassas, VA). Both cell lines were grown in RPMI medium containing 10% fetal bovine serum (FBS), 100 IU/mL penicillin, and 100 μg/mL streptomycin in a humidified incubator in 5% CO2 at 37°C. Passage of cell lines was performed at 1:3 dilution after detachment using sterile 0.05% trypsin-EDTA solution.

### IC50 calculations

Cultured cell lines were harvested and seeded into a 24-well plate (2 X10^4^ cells per well) in RPMI1640 culture medium and incubated for 24 hours at 37°C in a 5% CO2 incubator. The culture medium was then replaced with 100 uL of fresh medium containing varying concentrations of pemetrexed (0, 0.01, 0.1, 1, 10, 100μM) and incubated for 72 hours at 37°C in a 5% CO2 incubator. The IC50 assay was performed then performed using the MTT Cell Growth Assay Kit (Sigma-Aldrich, St. Louis, MO). Absorbance of the converted dye was measured using a Beckman DU-600 Spectrophotometer (Beckman Coulter Life Sciences, Indianapolis, IN) and data analyzed using the statistical software SPSS 19.0 (IBM, Chicago, USA).

### Mouse tumor xenograft modeling

Prior to *in vivo* animal modeling, approval was obtained by the Institutional Animal Care and Use Committee (IACUC) at the University of Pennsylvania. Human tumor-bearing murine xenografts were then created using two month-old female *nu*/*nu* mice (Crl: NUFoxnlnu, Charles River Laboratory, Wilmington, MA). Each mouse was injected subcutaneously in the flank with a suspension of H460 cells (1×10^7^) in sterile, endotoxin-free 50% Matrigel Matrix (Corning Inc., Corning, NY). When tumors reached a mean volume of approximately 200 mm^3^ (volume = π/6 × length × width × height), animals were randomized into treatment groups.

### *In vitro* analysis of thymidine salvage pathway activity

### ^3^H-thymidine assays

[^3^H]-thymidine (Perkin Elmer NET355001MC, PerkinElmer, Waltham, MA) was utilized for *in vitro* assessment of therapy-induced changes in thymidine salvage pathway activity in cultured human NSCLC cells. [^3^H]-thymidine specific activity was >10Ci(370GBq)/mmol and radiochemical purity >97%. H460 and H1299 cells were seeded (1×10^6^/well) in 6-well plate in RPMI1640 supplemented with 10% FBS and antibiotics, incubated 24 hours in 5% CO_2_ at 37°C. When cell cultures achieved 80% confluence, cells were exposed to treatment with either the vehicle (sterile water), pemetrexed (100 nM), or the combination of pemetrexed (100 nM) and cisplatin (10 μM) in growth media for varying exposure times ranging up to 48 hours. Drug-containing medium was then removed, and the cells were then washed and pulsed with 5 μCi [^3^H]-thymidine/well for 1 h. The cells were then washed and scraped into plastic vials. Scintillant (10 ml; Research Products International Corp., Mount Prospect, IL) was added to each vial and the radioactivity was counted on a scintillation counter (Beckman Coulter LS6500, Beckman Coulter Life Sciences, Indianapolis, IN). For ENT1 inhibition, cells were incubated with NBMPR (Nitrobenzylthioinosine) for 15min (100uM; Sigma-Aldrich, St. Louis, MO) prior to labeling with [^3^H]-thymidine.

### Immunoblotting

Therapy-induced changes in the protein expression of key components of the thymidine salvage pathway were assessed using Western blot analysis. H460 and H1299 cells were seeded (1×10^6^ cells/well) in 6-well plate in RPMI1640 supplemented with 10% FBS and antibiotics, incubated 24 h in 5% CO_2_ at 37°C. When cell cultures achieved 80% confluence, cells were exposed to treatment with either the vehicle (sterile water), pemetrexed (100 nM), or the combination of pemetrexed (100 nM) and cisplatin (10 μM) in growth media for varying exposure times ranging up to 48 hours. Whole cell lysates were then generated using a 1% Nonidet P-40 lysis buffer (Sigma-Aldrich Corp., St. Louis, MO). The suspension was homogenized by passages through a 20-gauge syringe needle and nuclear material removed through centrifugation at 14000 rpm for 15 min at 4°C. Cell lysates were then loaded onto a precast Nupage Bis-Tris Gels (invitrogen, Life Technologies, Corp., Grand Island, NY) for electrophoresis then transferred onto Hybond-P PVDF membranes (Sigma-Aldrich Corp., St. Louis, MO) for Western blot analysis. After blocking membranes with 5% non-fat milk in PBS with 0.1% Tween-20 buffer, PVDF membranes were probed using primary antibodies directed against human TK1 (Cell Signaling Technology, Danvers, MA; 1:5000), human TS (Cell Signaling Technology; 1:4000), human ENT1 (Abcam, Cambridge, MA; 1:200), or human β-actin (Sigma-Aldrich Corp., St. Louis, MO; 1:10000). Membrane were then washed and incubated (1:3000) with species-specific secondary antibodies, either anti-rabbit or anti-mouse, conjugated to horseradish peroxidase (GE Healthcare Life Science; 1:3000) for 1 h, the proteins were detected using the Immobilon ECL system (EMD Millipore, Billerica, MA) and quantified using Image J software available through the National Institutes of Health (https://imagej.nih.gov/ij/index.html).

### Immunofluorescence

Therapy-induced changes in the intracellular localization of ENT1 were assessed using immunofluorescence. Cells were fixed with 4% paraformaldehyde at room temperature for 10 min. Cells were washed with PBS, permeabilized and blocked with 1% BSA / 10% normal goat serum / 0.3M glycine in 0.1% PBS-Tween solution for 1 h. Cells were then incubated with a primary antibodies directed to human ENT1 (Abcam, Cambridge, MA; 1:100) overnight at 4°C. After incubation with the primary antibody, cells were washed with PBS and incubated with Cy™2 AffiniPure Donkey Anti-Rabbit IgG (Jackson ImmunoResearch Inc., West Grove, PA) with at room temperature for 1 h. DAPI (Thermo Fisher Scientific Inc., Grand Island, NY) was used to stain the cell nuclei at a concentration of 0.5 μg/ml. For negative staining control, primary antibody was omitted during the procedure. Digital images were acquired using a Nikon E600 microscope. The results of the membrane positive immunofluorescence of ENT1 were semiquantitatively assessed and scored as previously described [22]. Scoring criteria were defined as follows: 0 for negative (0% to 5% staining); 1 for weakly positive (5% to 20% staining); 2 for moderately positive (20% to 50% staining); 3 and 4 for strongly positive (50% to 75% and ≥ 75%, respectively).

### PET scanning and image analysis

#### Preclinical PET imaging

Baseline FLT-PET scans were performed on the day prior to therapy. Mice were then treated with either the vehicle control (i.p. sterile PBS) or combination therapy with pemetrexed (i.p. 100 mg/kg) and cisplatin (10 mg/kg). A post-therapy FLT-PET was then performed at varying time points following administration of therapy.

For the assessment tumor response to therapy, tumor-bearing animals were treated for a period of two weeks with 100 mg/kg pemetrexed (i.p. daily; days 1-5 and 8-12) and 10 mg/kg cisplatin (i.p. once weekly). During this treatment period, tumor volumes were estimated by external caliper measurements. After therapy/imaging completion, mice were euthanized with carbon dioxide inhalation.

After anesthesia with inhaled isofluorane in O_2_ (3% induction, 1.5% maintenance), mice were injected intravenously with 300-350 μCi of [18F]FLT then allowed to recover from the anesthesia during the 60-min uptake time allowed for radiotracer accumulation. At 60 min post-injection, mice were anesthetized and imaged for a 15-min static acquisition on the small animal PET scanner (A-PET, built in collaboration with Philips Medical Systems) located at the University of Pennsylvania Small Animal Imaging Facility.

#### Human PET imaging

IRB approval was obtained through our Institutional Review Board for use of FLT-PET/CT under an FDA IND in this clinical trial. An adult patient with unresectable non-small cell lung cancer (NSCLC) receiving therapy with pemetrexed and carboplatin was imaged with FLT-PET within 3 days prior to starting therapy and at 2 hours following the 1^st^ administration of intravenous infusion of the chemotherapeutic regimen. Human FLT-PET/CT was performed on the Philips Ingenuity TF scanner (Philips Medical Systems, at the Perelman Center for Advanced Medicine at the University of Pennsylvania. FLT-PET static images were obtained after a 60 minute uptake time following intravenous injection of 5mCi of [18F]FLT.

#### Image analysis

For preclinical PET imaging, after acquisition, the images were reconstructed with the manufacturer software and tumor volumes of [^18^F] FLT quantitated with the freely available Amide 3D software (LG Software Innovations), which allows for multiplanar analysis of the tumor volume. A region of interest (ROI) was traced around the tumor volume creating a 3D ROI that could be sculpted to the 3D tumor perimeter and visually inspected in the axial, coronal and sagittal planes. Statistical analysis of this 3D tumor uptake volume was then performed using Amide software including ROI volume (mm^3^), mean counts/pixel, max PET counts/pixel and standard deviation of tumor counts/pixel. PET values generated by the A-PET machine are absolute pixel counts. In order to control for slight differences in radiotracer administration and *in vivo* biood distribution, absolute tumor counts were normalized to flank muscle (tumor ROI pixel value/thigh muscle ROI pixel value).

For human PET imaging, SUV_MAX_ of the primary NSCLC was measured from axial PET/CT fusion images at baseline and post-therapeutic FLT-PET/CT using Philips IntelliSpace Portal (v5.0.2.40009, Philips Healthcare Nederland B.V., Netherlands) software. Post-therapeutic changes in tumor avidity for FLT-PET were then calculated.

### Statistics

Analysis for statistical significance between 2 or more groups was performed using student T tests or ANOVA as appropriate. Kruskal-Wallis was performed to compare scoring of different time points for Immunofloresence of ENT1. P-values greater than 0.05 were considered significant. All analyses were performed with SPSS 19.0 (IBM Corp., Armonk, NY).

## SUPPLEMENTARY MATERIALS FIGURES


