# Supplementary material for: Early detection of pemetrexed-induced inhibition of thymidylate synthase in non-small cell lung cancer with FLT-PET imaging
Source: Oncotarget. 2016 Sep 16;8(15):24213–23. doi: 10.18632/oncotarget.12085 (PMC5421841; doi:10.18632/oncotarget.12085)
Supplement: Supplementary file 1 [file oncotarget-08-24213-s001.pdf]

Early detection of pemetrexed-induced inhibition of thymidylate synthase in non-small cell lung cancer with FLT-PET imaging

Supplementary Material

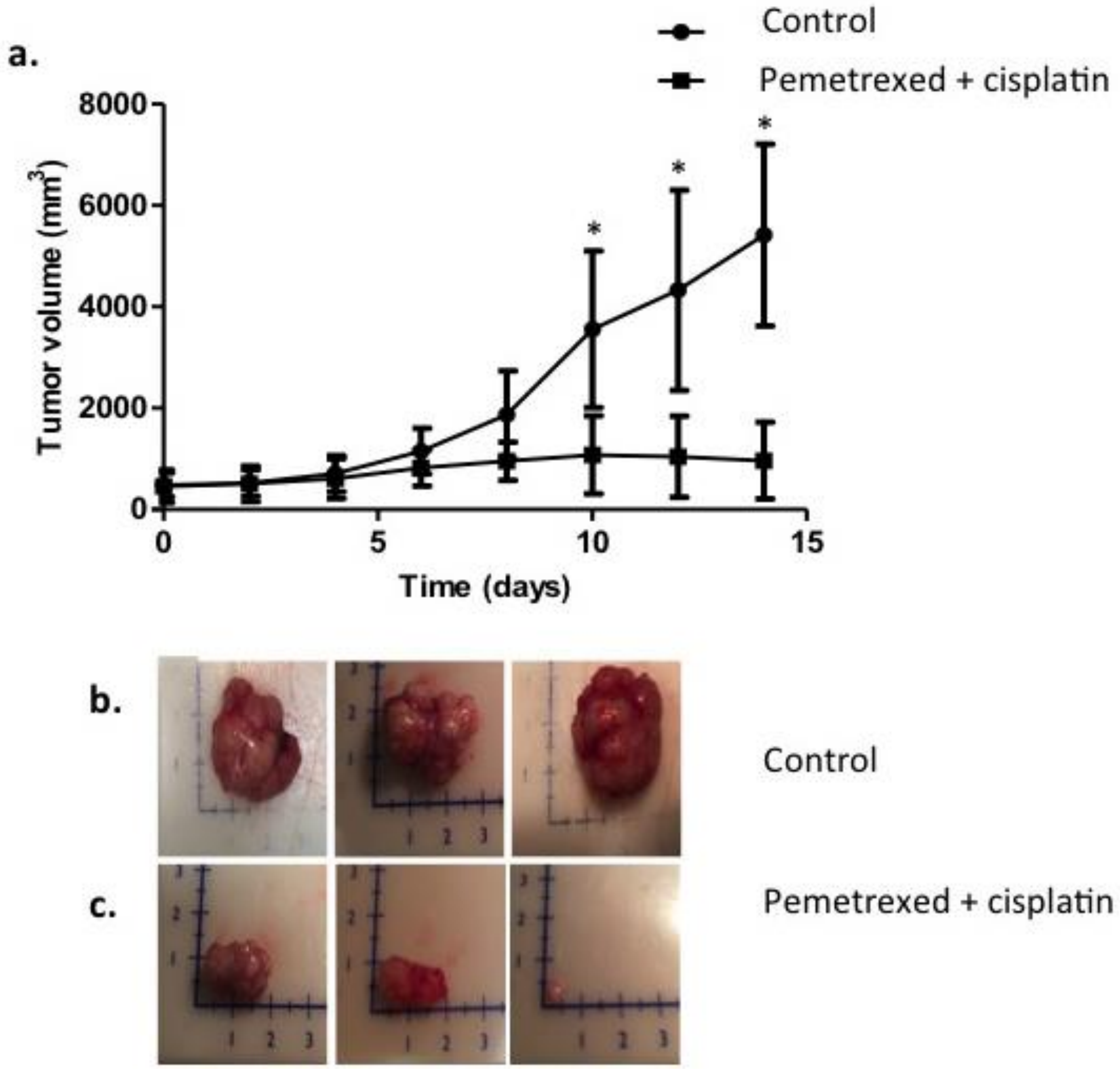

Supplemental Figure 1: Treatment of human NSCLC bearing xenografts with pemetrexed and cisplatin resulted in significant tumor growth inhibition. (a.) H460 bearing mouse xenografts were either treated as controls or given combination therapy with pemetrexed and cisplatin and tumors were measured over a 2 week course of therapy. (b., c.) Tumors were excised at necropsy demonstrating a significantly larger tumors in the (b.) control group relative to those (c.) treated with cisplatin and pemetrexed.

Cell lines	IC50 (µM)
H460	0.141
H1299	0.656
H1975	0.731
HCC827	0.175
H1650	0.182
H1703	0.618
H23	0.221
H1155	0.402

Supplemental Figure 2: IC50 calculations for pemetrexed sensitivity of NSCLC cell lines.

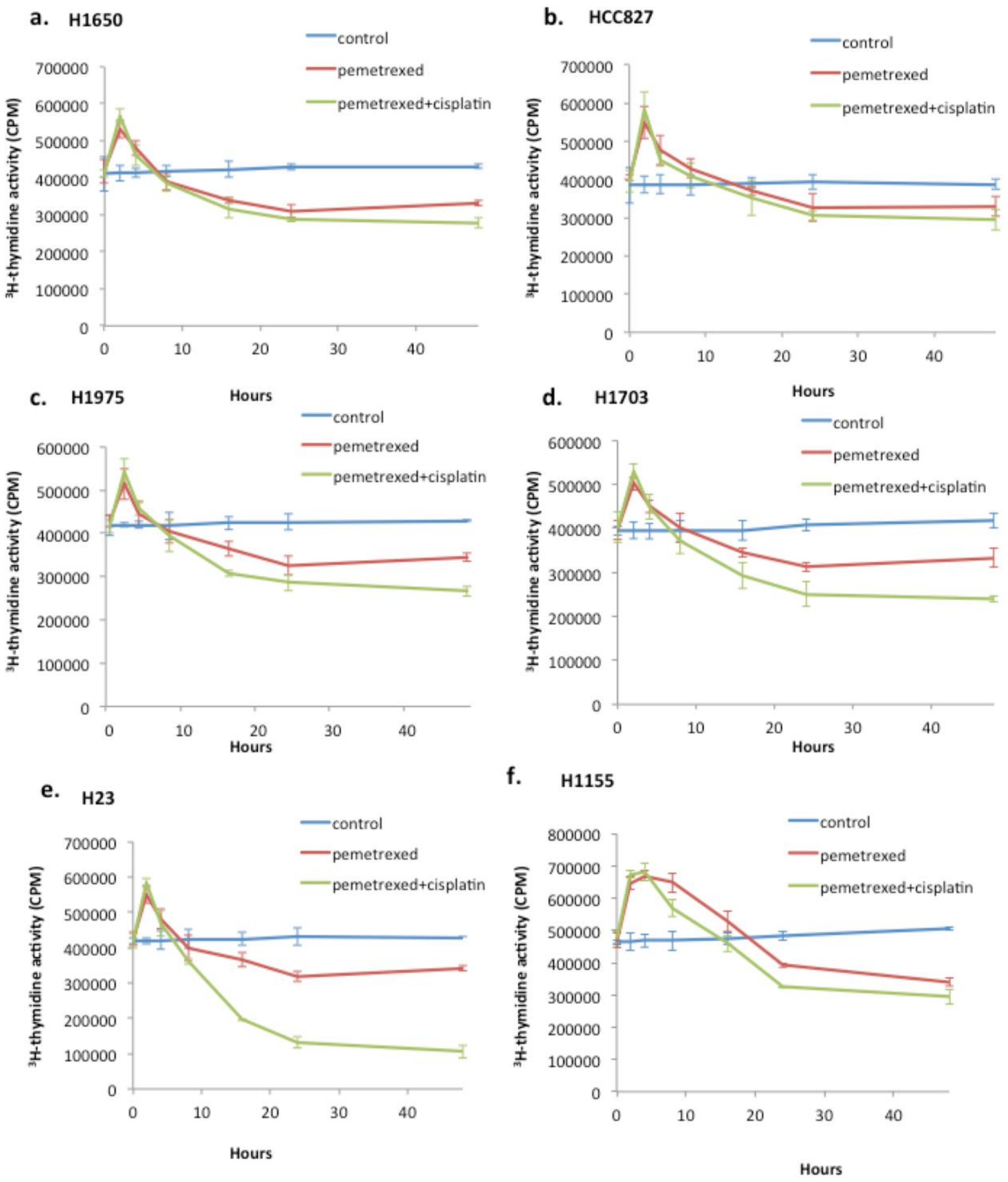

Supplemental Figure 3: Pemetrexed-induced TS inhibition results in a "flare" of the thymidine salvage pathway activity. 3H-thymidine assay was performed on PEM-sensitive NSCLC cell lines (a. -f.) in untreated control (culture medium only), pemetrexed (100nM) and combination therapy with pemetrexed (100nM) plus cisplatin (10mM). A "flare" in DNA salvage pathway activity was seen at 2 hrs of

exposure to pemetrexed in all 6 cell lines. Exposure to cisplatin showed no impact on the pemetrexed induced "flare".
